# Retraction: Implementation of Postpartum Hemorrhage Emergency Care Using a Bundle Approach at a Tertiary Care Hospital in North India

**DOI:** 10.7759/cureus.r64

**Published:** 2022-10-20

**Authors:** Singh Jigyasa, Sakshi Agrawal, Uma Pandey, Shikha Sachan, Mamta Rajan, Tej Bali Singh, Surabhi Sapna

**Affiliations:** 1 Obstetrics and Gynecology, Institute of Medical Sciences Banaras Hindu University (IMS BHU), Varanasi, IND; 2 Preventive Medicine, Institute of Medical Sciences Banaras Hindu University (IMS BHU), Varanasi, IND

This article has been retracted after an investigation was conducted by senior faculty and leadership at the Institute of Medical Sciences, Banaras Hindu University. This retraction is the result of a dispute between authors regarding authorship as well as the presence of incorrect and incomplete information. Dr. Uma Pandey initially contacted the journal with these concerns and, after an investigation by the Institute of Medical Sciences, Banaras Hindu University and failure among the authors to come to an agreement regarding amended authorship and corrections, we have elected to retract the article as suggested by Dr. Pandey and senior faculty and leadership at the Institute of Medical Sciences, Banaras Hindu University. Issues raised by Dr. Pandey and corroborated by the investigation include: missing authors, incorrect author placement for Dr. Pandey and others, and incorrect/incomplete information. Dr. Pandey and the journal regret that these disputes were not handled accordingly prior to submission.

